# Low-Level Laser Therapy in Dentistry: Biological Effects, Clinical Applications, and Future Perspectives

**DOI:** 10.7759/cureus.117988

**Published:** 2026-10-15

**Authors:** Akshay Katara, Rajendra Avhad, Saloni Kanodia, Vishwabharati I Tayade, Sameer Chauhan, Priyanka Bind, Pratik Surana

**Affiliations:** 1 Department of Dentistry, Omni Family Health, California, USA; 2 Department of Dentistry, Government Medical College, Ambernath, IND; 3 Department of Pedodontics and Preventive Dentistry, Faculty of Dental Sciences, SGT (Shree Guru Gobind Singh Tricentenary) University, Gurugram, IND; 4 Department of Conservative Dentistry and Endodontics, Guru Gobind Singh College of Dental Science and Research Centre, Burhanpur, IND; 5 Department of Prosthodontics and Crown Bridge, K. M. Shah Dental College and Hospital, Sumandeep Vidyapeeth (Deemed to be University), Vadodara, IND; 6 Department of Orthodontics and Dentofacial Orthopedics, Narsinhbhai Patel Dental College and Hospital, Sankalchand Patel University, Visnagar, IND; 7 Department of Pedodontics and Preventive Dentistry, Maitri College of Dentistry and Research Center, Durg, IND

**Keywords:** dental lasers, dentistry, laser dentistry, low level laser therapy, photobiomodulation, wound healing

## Abstract

Low-level laser therapy (LLLT), also referred to as photobiomodulation therapy (PBMT), is being integrated into dental treatment progressively. It has biological effects through light exposure, modulating cellular activity, helping to reduce pain, control inflammation, and heal tissue. LLLT has been demonstrated to have potential uses in periodontal therapy, endodontics, oral surgery, implantology, orthodontics, temporomandibular disorders (TMDs), and pediatric dentistry. However, considerable variations in laser parameters and treatment protocols limit the comparability of findings across studies and make it difficult to draw definitive conclusions. This review highlights the biological mechanisms, clinical application, current evidence, safety, limitations, and future perspectives of LLLT for dental purposes.

## Introduction and background

Modern dentistry is largely based on laser technology to achieve minimal invasiveness in both diagnostic and therapeutic procedures. LASER stands for Light Amplification by Stimulated Emission of Radiation. It is a source of electromagnetic radiation that emits light with specific properties such as coherence, monochromaticity, and directionality. These properties allow for the precise delivery of laser energy to targeted tissues [1].

Dental lasers can be classified broadly based on the power, wavelength, and the active medium. The power level classifies the lasers into high-power lasers, which produce primarily thermal and ablative effects; moderate-power lasers, with limited thermal effects; and low-level or cold lasers, which produce primarily photochemical effects rather than thermal effects. The latter are commonly referred to as photobiomodulation therapy (PBMT) or low-level laser therapy (LLLT) [2].

Wavelength, power density, and energy density are a few of the characteristics that determine how the body reacts to laser radiation, duration of exposure, and tissue characteristics [3]. Low-level laser irradiation is especially associated with cell photobiomodulation (PBM). It may affect mitochondrial activity, ATP production, inflammatory mediators, pain pathways, and tissue repair. Therefore, LLLT has received increasing attention in dentistry for its use in pain management, inflammation control, wound healing, periodontal therapy, endodontics, oral surgery, orthodontics, implantology, and temporomandibular disorders (TMDs) [1].

Dental lasers may have a wavelength within the visible, near-infrared, or infrared regions depending on the wavelength, and their active medium may be gaseous, liquid, solid-state, or semiconductor. Among them, semiconductor diode lasers are used frequently for low-level PBM due to their compact design and the possibility of delivering clinically relevant wavelengths [4,5].

With the increasing interest in minimally invasive and biologically oriented dental treatment, it is important to know the principles and biological effects of LLLT to be able to apply it in a clinically appropriate and evidence-based manner.

## Review

Type of review and methodology

This article is a narrative review of biological effects, clinical applications, safety, limitations, and future perspectives of LLLT/PBM in dentistry. The narrative review was conducted to provide an overview of the scope of the evidence and the focus of research in different areas of dentistry rather than to perform a quantitative analysis of the evidence.

The PubMed/MEDLINE, Scopus, Embase, Web of Science, and Google Scholar databases were searched for relevant studies. The search was not limited by date and was updated until September 2026. The search strategy consisted of combinations of terms related to LLLT and its applications in dentistry, such as “low-level laser therapy,” “photobiomodulation,” “PBM,” “dental lasers,” “dentistry,” “oral surgery,” “periodontology,” “endodontics,” “implantology,” “orthodontics,” “temporomandibular disorders,” and “pediatric dentistry,” along with Boolean operators and additional limits.

The inclusion criteria were the scope of research interested in the biological effects of low-level laser, its application in different fields of dentistry, treatment outcomes, safety, limitations, and future perspectives. The types of studies included original research, randomized controlled trials, systematic reviews, umbrella reviews, and relevant review articles. The exclusion criteria included the absence of the topic of lasers in dentistry, oral surgery, or periodontology. The references of the included articles were screened and reviewed for additional relevant literature. The narrative review was written based on the inclusion criteria and the organization of the retrieved evidence in terms of different areas of dentistry, biological effects of LLLT, treatment outcomes, safety, limitations, and future perspectives. It should be noted that the present study is a narrative review, and thus, the statistical analysis and pooling of data were not performed.

Biological effects of LLLT

Analgesic Effect

By influencing the activity of peripheral nerves and inflammatory reactions, LLLT has shown promise in alleviating dental pain. PBM may influence nerve conduction and nociceptor sensitivity while reducing the release of pain-associated inflammatory mediators. Alterations to prostaglandin synthesis, bradykinin and histamine release, pain thresholds, and endogenous endorphin generation are all potential pathways. Analgesia and enhanced patient comfort may result from the combined effects of these factors [6]. LLLT has been investigated as a primary or adjunctive approach for postoperative pain, endodontic procedures, orthodontic treatment, periodontal procedures, dental surgery, musculoskeletal pain, and TMDs. A meta-analysis by Díaz et al., which included 44 randomized clinical trials involving 1,816 participants, indicated that LLLT significantly reduced pain severity and improved mandibular function in patients with TMDs. Despite the wide range of laser parameters and treatment plans, the benefits were shown to be strongest for wavelengths between 810 and 940 nm. When properly adjusted, local low-dose radiation therapy (LLLT) can be an effective non-invasive method of pain management in the dental field [7].

Anti-inflammatory Effect

LLLT may exert anti-inflammatory effects by modulating cellular and molecular pathways involved in the inflammatory response. PBM can reduce the synthesis of cytokines and pro-inflammatory agents including interleukin-1β (IL-1β), tumor necrosis factor-α (TNF-α), and interleukin-6 (IL-6). It may also affect the function of inflammatory cells and localized swelling.

Subsequent to periodontal interventions including scaling, LLLT has also been linked to changes in prostaglandin activity and decreased production of matrix metalloproteinase-8 (MMP-8). Other growth factors influenced by PBM include platelet-derived growth factor (PDGF), transforming growth factor-beta (TGF-beta), and vascular endothelial growth factor (VEGF), which can support tissue repair and microvascular regeneration [8]. Both Zou et al. [9] and Morshedzadeh et al. [10] found that after PBM, clinical inflammatory markers like edema and crevicular fluid volume decreased, and there was also evidence of better vascular responses. These findings support the potential anti-inflammatory and tissue-healing effects of LLLT. However, the magnitude of the response may vary according to irradiation parameters, particularly wavelength and dose.

Enhanced Wound Healing

LLLT may promote wound healing by enhancing cellular proliferation, migration, collagen synthesis, angiogenesis, and tissue remodeling. PBM can stimulate reparative cells such as fibroblasts, keratinocytes, and endothelial cells and may modulate growth factors, including TGF-β and VEGF, thereby facilitating tissue repair [11]. There is clinical evidence supporting these effects in oral surgical and periodontal procedures. Pathak et al. [12] reported an association of 940-nm diode laser PBM after impacted third-molar extraction with reduced postoperative pain and improved mouth opening at follow-up. Correspondingly, in patients undergoing 890-nm PBM, Misra et al. [13] showed markedly improved wound healing after periodontal flap surgery, outperforming controls on the Landry Wound Healing Index at seven days and two weeks post-surgery. Reza et al. [14] also showed a significant increase in wound-healing scores at 3, 7, and 14 days after diode laser PBM using 810 and 660 nm wavelengths with an energy density of 6 J/cm^2^ and a significant decrease in pain scores during the first 72 hours. Moreover, Morshedzadeh et al. observed better healing of palatal wound following free gingival graft surgery with 940-nm PBM [10]. Together, these findings suggest that LLLT may be a useful adjunct to promote oral soft-tissue healing, but variations in wavelength, dose, irradiation site, and treatment protocol are important considerations.

Potential Antimicrobial Effects

LLLT may also have potential antimicrobial and antibiofilm effects, but these effects seem to be strongly dependent on the wavelength, dose, exposure conditions, and microbial species. PBM might directly affect microbial viability and biofilm behavior or enhance host defense mechanisms and tissue healing. However, conventional LLLT is not mainly designed to induce microbial destruction in comparison with antimicrobial photodynamic therapy. Therefore, to establish uniform techniques and clinical importance, additional controlled trials are needed, and its antibacterial effects should be seen as supplementary and perhaps variable [15].

Modulation of Immune Responses

LLLT can modulate immune responses by affecting the activity and function of various immune cells. PBM has been shown to increase the activity of macrophages and lymphocytes and to promote phagocytic activity, thus supporting local host defense and tissue repair. It may also influence T-cell activity, immunoglobulin production, cytokine signaling, and the complement system. Such immunomodulatory effects may be beneficial to modulate excessive inflammation while supporting the normal healing response. However, the magnitude and direction of the immune modulation depend on wavelength, dose, irradiance, and treatment duration [1].

Application of LLLT in dentistry

Application of LLLT in Periodontology

LLLT is an adjunctive therapy in periodontology being increasingly studied for its anti-inflammatory, analgesic, regenerative, and wound-healing properties. It may modulate periodontal inflammation and stimulate fibroblast activity, collagen synthesis, angiogenesis, and tissue repair. Bhardwaj et al. [16] in a case report described the use of LLLT in combination with bovine-derived demineralized bone matrix for the treatment of intraosseous periodontal defects. However, this evidence should be received with caution as it comes from a single case study, which cannot provide sufficient support for the general efficacy of the treatment. Kohale et al. [17] found better wound healing following scalpel gingivectomy, and Gündoğar et al. [18] reported better clinical periodontal parameters when LLLT was combined with non-surgical periodontal therapy. Together, these results suggest that LLLT may improve periodontal healing and treatment results, but a limiting factor is the difference in laser parameters and treatment protocols.

Trigeminal Neuralgia

Recurring, unilateral, transient, electric shock-like pain along the course of one or more trigeminal nerve branches characterizes TN, a severe neuropathic pain disease. LLLT has been investigated as a non-invasive adjunctive modality for managing pain associated with trigeminal neuralgia [19]. Walker [20] treated 26 patients using a low-power He-Ne laser at a wavelength of 632.5 nm for a variety of painful disorders, including TN. The majority of patients saw a significant reduction in pain frequency and intensity after treatment. The results show that LLLT may alleviate neuropathic pain, but the results should not be interpreted too hastily due to the small sample size and the inclusion of many pain types [20].

TMDs

TMDs are a group of conditions that cause pain and dysfunction in the temporomandibular joint and the muscles of mastication and surrounding tissues. The most common symptoms of TMDs are pain, joint sounds, and limitation of mandibular movement. LLLT has been studied as a non-invasive adjunct in the management of TMD due to its analgesic, anti-inflammatory, and tissue repair properties. However, clinical results are still inconsistent due to variations in wavelength, dose, site of application, and treatment protocols [19]. Kulekcioglu et al. [21] studied 35 individuals in a randomized, controlled trial and discovered that both the LLLT and placebo groups saw a considerable decrease in pain. However, when it came to the number of tender sites, lateral mandibular movement, and mouth opening, only the LLLT group showed a substantial improvement. The results suggest that LLLT could have additional functional benefits for TMD treatment, but more high-quality clinical trials and standardized treatment regimens are required.

Application of LLLT in Pediatric Dentistry

LLLT can be applied in pediatric dentistry because of its analgesic, anti-inflammatory, and tissue healing effects and non-invasive nature [22,23]. PBM has also been investigated as an adjunct to pulpotomy in primary teeth. Yavagal et al. [22] conducted a comparative study between laser photobiomodulation (LPBM) pulpotomy and conventional formocresol pulpotomy in primary molars. The results showed a significantly higher radiographic success rate at nine months in the LPBM group. These results suggest that LPBM may be a promising alternative approach for pulpotomy of primary teeth. However, additional long-term clinical studies are required to determine its efficacy and standardized treatment parameters in the pediatric population. LLLT has potential applications in pediatric dentistry due to its analgesic, anti-inflammatory, and tissue-healing properties and its non-invasive nature. PBM has also been investigated as an adjunct to pulpotomy in primary teeth. 

Application of LLLT in Orthodontics

LLLT is introduced into orthodontic practice as a non-invasive adjunctive modality to facilitate orthodontic tooth movement and to enhance patient comfort. PBM can affect cell activity and bone remodeling around teeth in motion, which can aid in tooth movement while minimizing orthodontic discomfort and pain. It has also been studied for its potential effect on orthodontically induced root resorption. When low-energy parameters are properly selected, LLLT is designed to generate photochemical effects without a significant thermal elevation of the target tissue. Nevertheless, possible explanations for discrepancies in reported results include variations in wavelength, dose, and treatment regimens [24].

Application of LLLT in Endodontics

LLLT is mainly used as an adjunctive therapy in endodontics due to its ability to modulate tissues, reduce inflammation, and alleviate pain. It may help to reduce postoperative pain and inflammation after root canal treatment and may help to heal periapical tissue. PBM's antimicrobial potential and its effects on bone and tissue repair have also been investigated. Pereira et al. in an umbrella review reported that LLLT might add benefits when used in addition to root canal treatment and retreatment, but due to variations in laser parameters and clinical protocols, no definitive conclusions can be drawn [15].

Application of LLLT in Implantology

In implantology, LLLT has been studied for its ability to speed up soft tissue healing, decrease postoperative pain and inflammation, and encourage osseointegration. PBM might boost cellular activity, the process of neovascularization, and wound healing around the implant site. Research by Reza et al. demonstrated that PBM can alleviate postoperative pain and speed wound healing following dental implant surgery. The use of LLLT as a supplement to implant operations to speed up tissue recovery is similar. However, there is conflicting evidence regarding its effects on osseointegration and long-term outcomes of the implant, highlighting the need for standardization of treatment protocols [14].

Safety and limitations

In general, LLLT is safe when appropriate laser parameters are used. It is non-thermal and therefore causes minimal tissue damage. Protective eyewear should be used, and appropriate dose selection is important. Modifications in wavelength, energy density, treatment time, and irradiation technique are the main constraints. Inconsistencies in study designs, inadequate sample sizes, and a lack of long-term data further hinder the development of consistent clinical guidelines.

Future perspectives

In the future, LLLT applications will probably involve personalized treatment protocols, better laser technologies, and integration with digital dentistry and regenerative therapies. Artificial intelligence may also prove helpful in the optimization of the laser parameters according to the individual patient and treatment characteristics. Well-designed clinical trials are essential to provide uniform protocols and long-term clinical advantages.

## Conclusions

LLLT is a promising non-invasive adjunctive modality in dentistry, with potential analgesic, anti-inflammatory, regenerative, and wound-healing effects. It has been found useful in various dental specialties, but differences in treatment parameters and clinical protocols prevent definitive conclusions. Its role in normal dental practice is yet to be determined through rigorous clinical trials and established standards.

## References

[1] Asnaashari M, Safavi N (2013). Application of low level lasers in dentistry (endodontic). J Lasers Med Sci.

[2] Grzech-Leśniak K (2026). Lasers in dentistry—overview based on FDI policy statement. Int Dent J.

[3] Dompe C, Moncrieff L, Matys J (2020). Photobiomodulation-underlying mechanism and clinical applications. J Clin Med.

[4] Luke AM, Mathew S, Altawash MM, Madan BM (2019). Lasers: a review with their applications in oral medicine. J Lasers Med Sci.

[5] Rathod A, Jaiswal P, Bajaj P, Kale B, Masurkar D (2022). Implementation of low-level laser therapy in dentistry: a review. Cureus.

[6] Naseri M, Asnaashari M, Moghaddas E, Vatankhah MR (2020). Effect of low-level laser therapy with different locations of irradiation on postoperative endodontic pain in patients with symptomatic irreversible pulpitis: a double-blind randomized controlled trial. J Lasers Med Sci.

[7] Díaz L, Restelli L, Valencia E, Atalay DI, Abarca JM, Gil AC, Fernández E (2025). Effectiveness of low-level laser therapy on temporomandibular disorders. A systematic review of randomized clinical trials. Photodiagnosis Photodyn Ther.

[8] Gil AC, Díaz L, Von Marttens A (2025). The efficacy of low-level laser therapy in oral surgery: a systematic review of randomized controlled trials. Photodiagnosis Photodyn Ther.

[9] Zou Q, Zhang S, Jiang C, Xiao S, Wang Y, Wen B (2024). Low-level laser therapy on soft tissue healing after implantation: a randomized controlled trial. BMC Oral Health.

[10] Morshedzadeh G, Aslroosta H, Vafaei M (2022). Effect of GaAlAs 940 nm photobiomodulation on palatal wound healing after free gingival graft surgery: a split mouth randomized controlled clinical trial. BMC Oral Health.

[11] Gopal L, Palwankar P, Dhalla N (2024). The efficacy of low-level laser therapy on the healing of oral wounds: a systematic review. Cureus.

[12] Pathak R, Kale TP, Pattar V, Garg A, Srivastava A (2026). The effectiveness of photobiomodulation using 940-nm diode laser for reducing pain, swelling and trismus after third molar surgery: a single-blinded randomized control trial. J Maxillofac Oral Surg.

[13] Misra P, Kalsi R, Anand Arora S, Singh KS, Athar S, Saini A (2023). Effect of low-level laser therapy on early wound healing and levels of inflammatory mediators in gingival crevicular fluid following open flap debridement. Cureus.

[14] Reza B, Soheil N, Ehsan B, Kourosh S, Reza F (2021). Efficacy of photo bio-modulation therapy for pain relief and soft tissue wound healing after dental implant surgery: a double-blind randomized clinical trial. J Photochem Photobiol.

[15] Pereira BS, Vieira WA, do Nascimento Santana V, Braido A, Ribeiro MAG (2025). Efficacy of low-level laser as an adjunctive therapy in root canal treatment and retreatment: an umbrella review. Lasers Med Sci.

[16] Bhardwaj S, George JP, Remigus D, Khanna D (2016). Low level laser therapy in the treatment of intra-osseous defect - a case report. J Clin Diagn Res.

[17] Kohale BR, Agrawal AA, Raut CP (2018). Effect of low-level laser therapy on wound healing and patients' response after scalpel gingivectomy: a randomized clinical split-mouth study. J Indian Soc Periodontol.

[18] Gündoğar H, Şenyurt SZ, Erciyas K, Yalım M, Üstün K (2016). The effect of low-level laser therapy on non-surgical periodontal treatment: a randomized controlled, single-blind, split-mouth clinical trial. Lasers Med Sci.

[19] Nadhreen AA, Alamoudi NM, Elkhodary HM (2019). Low-level laser therapy in dentistry: extra-oral applications. Niger J Clin Pract.

[20] Walker J (1983). Relief from chronic pain by low power laser irradiation. Neurosci Lett.

[21] Kulekcioglu S, Sivrioglu K, Ozcan O, Parlak M (2003). Effectiveness of low-level laser therapy in temporomandibular disorder. Scand J Rheumatol.

[22] Yavagal CM, Lal A, Patil VVC, Yavagal PC, Neelakantappa KK, Hariharan M (2021). Efficacy of laser photobiomodulation pulpotomy in human primary teeth: a randomized controlled trial. J Indian Soc Pedod Prev Dent.

[23] Singh S, Chakraborty A, Saju AR, Singh R, Sen A, Shrinivas S, Surana P (2024). Comprehensive review on low-level laser therapy in dentistry. J Pharm Bioallied Sci.

[24] Chintavalakorn R, Saengfai NN, Sipiyaruk K (2022). The protocol of low-level laser therapy in orthodontic practice: a scoping review of literature. J Int Soc Prev Community Dent.

